# Tacrolimus Maintains the Balance of Neutrophil Extracellular Traps by Inducing DNA Methylation of Neutrophils to Reduce Immune Rejection

**DOI:** 10.3390/life13122253

**Published:** 2023-11-25

**Authors:** Liang Xu, Ming Cai

**Affiliations:** Department of Urology, The Third Medical Center of Chinese PLA General Hospital, Medical School of Chinese PLA, Beijing 100853, China; xul351@sina.com

**Keywords:** tacrolimus (FK506), PMA, NETs, DNA methylation, neutrophils

## Abstract

Immune rejection is a significant concern in organ transplantation, as it can lead to damage to and failure of the transplanted organ. To prevent or treat immune rejection, transplant recipients are commonly administered immunosuppressive drugs. Tacrolimus (FK506) is a widely used immunosuppressive drug in organ transplantation. The excessive formation of neutrophil extracellular traps (NETs) can contribute to inflammation and tissue damage. Although NETs play an antimicrobial role, their overproduction can be harmful. To investigate the mechanism by which FK506 suppresses immune rejection, we utilized HL-60 cells, which were differentiated into neutrophils using DMSO and induced to form NETs with phorbol myristate acetate (PMA), a very efficient and frequently used drug for inducing NET formation. By comparing pre- and post-treatment with FK506, we examined whether FK506 affects the formation of NETs. Various experimental techniques were employed, including confocal imaging for visualizing cell NETs, qPCR and Western blotting for gene and protein expression analyses, ELISAs for protein content detection, and LC-MS/MS for methylation detection. In our study, we discovered that FK506 can enhance DNA methylation, which likely contributes to the reduction in NETs. Genes and proteins related to methylation, namely, DNMT3B and TET3, exhibited significant correlations with methylation. Consistent changes in both genes and proteins suggest that DNMT3B and TET3 are key factors that are influenced by FK506, resulting in enhanced DNA methylation and the potential inhibition of PMA-induced NET production. In summary, we have identified a novel mechanism by which FK506 inhibits NET production through the enhancement of DNA methylation. This finding highlights a new aspect of FK506′s immunosuppressive effect. Our results provide valuable insights for clinical research, immunosuppression, and organ preservation strategies.

## 1. Introduction

Immune rejection is a major concern in organ transplantation, as it can occur when a recipient’s immune system recognizes the transplanted organ as foreign and initiates an immune response against it. This immune response can cause damage to the transplanted organ and, ultimately, lead to its failure [1]. To prevent or treat immune rejection, transplant recipients are typically administered immunosuppressive drugs to inhibit the immune system and prevent it from attacking the transplanted organ [2,3,4]. Tacrolimus, also known as FK506, is an immunosuppressive drug that is commonly used to prevent or treat immune rejection in organ transplantation [5,6,7,8]. By inhibiting calcineurin, FK506 reduces the production of cytokines, which are proteins that mediate inflammation and immune responses, thus suppressing the immune system and preventing it from attacking the transplanted organs [9,10,11].

Neutrophils, a type of white blood cell, play a crucial role in immune responses to infection and inflammation. They were first discovered in 1996 by Takei et al. [12]. These cells are known for their ability to kill bacteria and other pathogens, and they do this through several mechanisms, including the formation of NETs [13,14,15,16,17]. Neutrophils and NETs play a dual role in host homeostasis, offering protection against infectious diseases while also contributing to pathological changes, particularly in autoimmune and autoimmune diseases [18]. While neutrophils play a role in the immune response, their involvement in immune rejection in organ transplantation remains inadequately understood. Some research suggests that neutrophils may be involved in the early stages of acute rejection, which occurs within weeks to months after transplantation when the immune system recognizes and attacks the transplanted organ [19,20,21]. However, the precise mechanisms by which neutrophils contribute to immune rejection are still the subject of ongoing research and lack full comprehension. In organ transplantation, immune rejection is typically prevented or treated with immunosuppressive drugs, such as FK506, cyclosporine, and others, which suppress the immune system and prevent it from attacking the transplanted organ [22,23,24]. FK506, which is commonly used for this purpose, functions by inhibiting the activity of calcineurin, an enzyme involved in the activation of immune cells. This action effectively suppresses the immune system, thus safeguarding the transplanted organs [25,26]. Notably, there is no evidence to suggest that FK506 exerts a direct effect on NET formation or functions.

To elucidate the mechanism underlying the immunosuppressive action of FK506, we conducted experiments utilizing HL-60 cells. These cells were induced to differentiate into neutrophils and subsequently stimulated to generate NETs with PMA. By comparing the cellular responses before and after the administration of FK506, we probed the impact of FK506 on the formation of NETs. Our results indicate that FK506 leads to an increase in DNA methylation, potentially contributing to the reduction in NETs. Further research is necessary to fully comprehend the relationship between DNA methylation, NET formation, and immune rejection in organ transplantation. Additionally, future studies should explore the therapeutic potential of drugs that modulate DNA methylation or NET formation.

## 2. Materials and Methods

### 2.1. Reagents

PMA (CAS: 16561-29-8) was purchased from Solarbio Life Sciences (Beijing, China), Tacrolimus (FK506) (CAS: 104987-11-3) was purchased from Selleck (Selleck Chemicals, Houston, TX, USA), and DMSO was purchased from Sinopharm Chemical Reagent Co., Ltd. (Beijing, China). LC MS/MS standards of cytidine (C) (CAS: 565-46-3), 5-methylcytosine (5mC) (CAS: 554-01-8), thymidine (CAS: 50-89-5), S-adenosylmethionine (SAM) (CAS: 17176-17-9), and S-adenosylhomocysteine (SAH) (CAS: 979-92-0) were purchased from Shanghai Yuanye Bio-Technology Co., Ltd. (Shanghai, China). Methanol and acetonitrile (LC grade) were purchased from the Fisher Company (Fisher, Hampton, NH, USA).

### 2.2. Cell Lines, Culture, and Treatment

One human HL-60 cell line was obtained from Otwo Biotech (Shenzhen) Inc. (Shenzhen, China). All cells were grown in 89% RPMI 1640 medium (Gibco, ThermoFisher Scientific, Waltham, MA, USA) with 10% FBS at 37 °C in a 5% CO_2_ atmosphere.

HL-60 cells were incubated for 2 days at 37 °C in a 5% CO_2_ incubator. Neutrophils were induced after 3 days of treatment with 1.25% DMSO and then treated with PMA and FK506. In the first group, 100 μL of DMSO was added to the control group (DMSO). The second group was the PMA treatment group (PMA) in which 100 nmol/L of PMA (final concentration) was added to stimulate and induce the generation of NETs at 37 °C. The third group was the FK506 treatment group, which was treated with the addition of 20 ng/mL of FK506 for 3 h. The fourth group was the combined treatment group (PMA + FK506), in which PMA and FK506 at their original concentrations were added simultaneously. Each group had three independent repetitions (n = 3). The cells were collected after treatment and then subjected to the following experiments and tests. PMA and FK506 were dissolved in DMSO.

### 2.3. Cell Viability via MTT Assays, NET Visualization Using Confocal Microscopy

HL-60 cells were seeded into 8-well plates (5 × 10^5^ cells per well) and stimulated with 100 nM of PMA, 20 ng/mL of FK506, and the combined treatment group (PMA + FK506 concentrations of 100 nM and 20 ng/mL, respectively). The medium was removed and cells were then incubated with 100 μL of a 3-(4,5-dimethylthiazol-2-yl)-2,5-diphenyl tetrazolium bromide (MTT, Sigma, Ronkonkoma, NY, USA) solution (5 mg/mL) for 2–4 h. After discarding the medium, 100 μL DMSO was added and gently mixed for 10 min. Finally, the absorbance at 570 nm was measured by an MD microplate reader (Molecular Devices, San Jose, CA, USA). Three replicates per condition were assayed, and the average values from three to five separate experiments are presented. Data of cell viability are expressed as a percentage of the control (DMSO group). After 3 h, 1 μM of SYTOX Green (SYTOX™ Green Nucleic Acid Stain—5 mM of solution in DMSO, S7020, Invitrogen™, Thermo Fisher Scientific Inc., Waltham, MA, USA) was added for 10 min, and the DNA of compromised cell membranes was visualized using a Leica confocal microscope (Leica, TCS SP8, Wetzlar, Germany) equipped with 40× and 10× magnification objectives and measured via a fluorometer with an excitation/emission of 485/518 nm, respectively [27,28,29].

### 2.4. Extraction of DNA and RNA from Cells

DNA extraction was performed using a cell DNA extraction kit (D1001, SinoGene Scientific Co., Ltd., Beijing, China). RNA extraction was conducted using TRIzol Reagent (TransZol Up, ET111-01, TransGen Biotech Co., Ltd., Beijing, China). DNA and RNA were extracted according to the manufacturer’s instructions, and their concentration and purity were detected via Nanodrop. Samples with OD260/280 at 1.8–2.0 were used for subsequent experiments.

### 2.5. Detection of NETosis, DNMTs, and TETs via the ELISA Method

NETosis and DNMTs were detected via the ELISA method using a NETosis Assay Kit (ab235979, Abcam, Cambridge, UK) and a DNMT Activity Assay Kit (Colorimetric) (ab113467, Abcam, Cambridge, UK), respectively. DNMT1, DNMT3A, DNMT3B, TET1, TET2, and TET3 ELISA kits were purchased from Jiangsu Jingmei Biological Technology Co., Ltd. (Suzhou, China). All tests were carried out according to the manufacturer’s instructions, and MD microplate readers (Molecular Devices, USA) were used for testing.

### 2.6. Western Blotting (WB) to Detect TET1 and DNMT1 Protein Expression

HL-60 cells were lysed in RIPA buffer, and the total protein concentration was measured using Bradford kits (Jiangsu Jingmei Biological Technology Co., Ltd., China). GAPDH (ab8245, Abcam, Cambridge, UK), TET1 (DF6428, Affinity Biosciences, Cambridg, UK), and DNMT1 (DF7376, Affinity Biosciences, UK) antibodies were used. For detailed instructions, please refer to the study of Mroczek et al. [29].

### 2.7. Gene Expression Analysis of DNA Methylation and NET-Related Genes via qPCR

Total RNA samples were transcribed into cDNA (42 °C for 10 min, 65 °C for 10 s, and stored at 4 °C) via a High-Capacity RT-PCR Kit (TransScript^®^ II All-in-One First-Strand cDNA Synthesis SuperMix for qPCR (One-Step gDNA Removal), TransGen Biotech Co., Ltd., Beijing, China) according to the manufacturer’s protocol. Primers were designed via PrimerPremier 6.0 software and sent to Primer Synthesis Company (Beijing Tsingke Biotechnology Co., Ltd., Beijing, China) for synthesis. The primers of the evaluated genes are outlined in Table 1, and GAPDH was used as the reference gene to ensure equal loading. Quantitative real-time qPCR was performed using 96-well microwell plates with a total volume of 20 μL, containing 1 μL of template cDNA (10 ng/μL), 0.5 μL of forward primer (10 μM), 0.5 μL of reverse primer (10 μM), and 10 μL of TB Green^®^ Fast qPCR Mix (Code No. RR430S, Takara Biomedical Technology Co., Ltd., Beijing, China). qPCR reactions were performed at 95 °C for 3 min, followed by 40 cycles of 95 °C for 10 s and 60 °C for 30 s using two-step qPCR. All qPCR reactions were performed using a CFX96™ Real-Time PCR detection system (Bio-Rad, Hercules, CA, USA) and measured in triplicate to ensure methodological reproducibility.

### 2.8. 5mC Detection of Total DNA via LC MS/MS

According to the methods of Friso et al. [30], 1 µg of DNA was denatured by heating at 100 °C for 3 min and subsequently chilled in a refrigerator at 4 °C for 10 min. A one-tenth volume of 0.1 M of ammonium acetate (pH 7.5) and two units of DNase I (NEB, Ipswich, MA, USA) were added. The mixture was then incubated at 37 °C for 3 h. Two units of alkaline phosphatase were subsequently added to the solution (NEB, USA). Incubation was continued for an additional 3 h at 37 °C. Afterward, the mixture was incubated overnight at 37 °C with 40 units of exonuclease I (Takara Biomedical Technology Co., Ltd., Beijing, China). The complete lysis mixture was placed in a refrigerator at 4 °C for LC MS/MS detection [31].

The hydrolyzed DNA was analyzed via liquid chromatography–electrospray ionization–tandem mass spectrometry with multiple-reaction monitoring (LC-ESI-MS/MS-MRM, XEVO TQS, Waters, Milford, MA, USA) for both test samples and standards. Thymidine was used as the internal standard (IS). 5mC and cytidine (C) were identified and quantified using UPLC equipped with a triple quadrupole (QqQ) mass spectrometer. A volume of 5 μL of the hydrolyzed DNA (25 ng) was injected into a UPLC-BEH amide column (Waters, USA) for chromatographic separation, using water with 0.1% formic acid (A) and ACN with 0.1% formic acid (B) as mobile phases. A flow of 95% A and 5% B was maintained for 10 min. The column was operated at 30 °C, with a flow rate of 0.4 mL/min. The triple quadrupole parameters were optimized by selecting positive-ion polarity for the system. The operating conditions were as follows: gas temperature, 400 °C; drying nitrogen gas, 9 L/min; nebulizer pressure, 40 psi; sheath gas temperature, 350 °C; sheath gas flow, 10 L/min; capillary voltage, 3500 V; nozzle voltage, 1000 V. These conditions were used for nucleotide analysis with a scan time of 50 ms. The multiple-reaction monitoring (MRM) method examines three transitions for each analysis (see Table 2). The total amount of 5mC in test samples was calculated from the 5mC MRM peak area divided by the sum of 5mC and cytidine (C) peak areas (5mC/C).

### 2.9. SAM and SAH Detection in Cells via LC MS/MS

SAM and SAH were quantified via LC MS/MS as described previously [32], with minor modifications to run on UPLC coupled to a mass spectrometer. Cell samples were washed twice with cold PBS and subsequently subjected to bead beating in a mixture of 80% methanol/water (LC-MS-grade methanol, Fisher Scientific) at −20 °C. The extraction mixture was vortexed for 10 s, subjected to ultrasonication for 30 min, and centrifuged for 15 min at 12,000× *g*/min. The supernatants were transferred to an autosampler vial, and 5 µL of the mixture was then used for UPLC-MS/MS analysis (UPLC I Class, XEVO TQS, Waters, USA). Separation was performed on a BEH amide column (130 Å, 1.7 µm, 1 mm × 100 mm, 1/pkg, Waters, USA) for nucleosides. The mobile phases consisted of (A) 100% water containing 0.1% formic acid and (B) 100% acetonitrile containing 0.1% formic acid. The following gradient was applied: 0–1.0 min, 95% B; 1.0–3.0 min, 30% B; 3.0–3.1, 95% B; 3.1–5 min, 95% B. The mass spectrometer was operated in positive-ion mode using the following settings: capillary voltage, 2.5 kV; source temperature, 150 °C; desolvation temperature, 350 °C; cone gas flow rate, 50 L/h; and desolvation gas flow rate, 650 L/h. The scan dwell time was set to 0.2 s for both analytes. The optimized MRM ion source parameters are presented in Table 3.

### 2.10. Data Processing and Drawing

A *t*-test was performed to calculate the different *p*-values, a histogram was plotted using GraphPad Prism 8.8, and correlation analysis was performed using the R language (Version 4.3.0). Data are expressed as means ± SE.

## 3. Results

### 3.1. FK506 Significantly Increased DNA Concentrations and ROS Levels but Decreased NET and NETosis Levels after PMA Treatment in Cells

**Figure 1:**
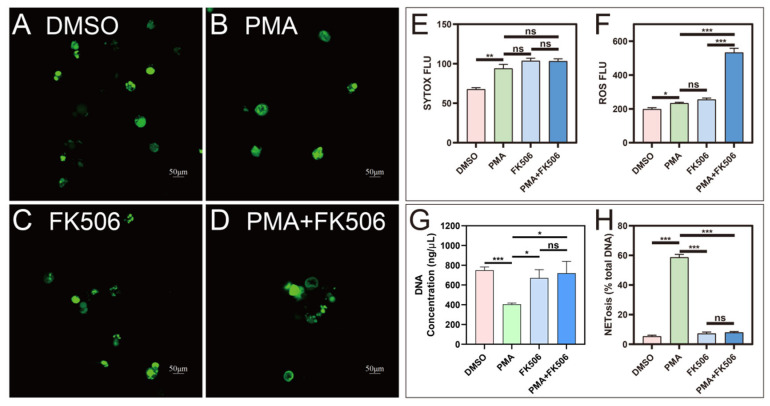
Confocal images of NETs and the changes in ROS, DNA, and NETosis levels in four groups of cells. (**A**–**D**) Confocal images of DMSO, PMA, FK506, and PMA + FK506 groups of cells, respectively. (**E**) SYTOX FLU (fluorescence value). (**F**) ROS FLUX (fluorescence value). (**G**) Intra-cellular DNA concentration. (**H**) NETosis percentage in cells. ns indicates no significant difference, * indicates *p* < 0.05, ** indicates *p* < 0.01, *** indicates *p* < 0.001. n = 3.

Confocal imaging of SYTOX-stained HL-60 cells revealed a substantial increase in the fluorescence intensity of SYTOX (Figure 1A–D) after PMA treatment compared to the control (DMSO, Figure 1A), signifying the formation of neutrophil extracellular traps (NETs; Figure 1B). Notably, treatment with FK506 alone (Figure 1C) or in combination with PMA (Figure 1D) did not exhibit a significant variance in SYTOX fluorescence intensity when compared to the PMA group (Figure 1E). The cell viability was assessed using the MTT method concurrently with the confocal imaging of SYTOX-stained HL-60 cells. The results indicated a decrease in cell viability following PMA treatment. However, no significant difference was observed between the treatments of FK506 alone and PMA + FK506 (see Supplementary Data, Figure S1). Additionally, there was a noteworthy surge in the levels of reactive oxygen species (ROS) following PMA treatment. While there was no marked difference between FK506 and PMA treatments, the combination of PMA and FK506 further accentuated the elevation in ROS levels, reaching a 2.28-fold increase compared to the PMA group (Figure 1F). An analysis of the DNA content in cells extracted from the four treatment groups (DMSO, PMA, FK506, and PMA + FK506) unveiled significant alterations. The DNA content experienced a marked decrease after PMA treatment but returned to baseline levels following FK506 treatment. Treatment with PMA + FK506 effectively maintained a stable DNA content (Figure 1G). This suggests that FK506 aids in retaining DNA within cells, thereby mitigating DNA leakage and NET formation. A subsequent investigation utilizing FK506 to compare the two treatment groups elucidated the mechanisms of PMA-induced NETosis. The percentage of NETosis substantially diminished from 58.7% to 7.9%, a difference not significantly distinct from the control group (5.3%) (Figure 1H). These findings conclusively demonstrate that FK506 effectively reduces the percentage of NETosis. Furthermore, after treatment with PMA + FK506, the level of NETosis exhibited no significant difference from that of the control group (DMSO) (Figure 1H), providing further confirmation of the inhibitory effect of FK506 on NET formation.

### 3.2. FK506 Significantly Increased the Level of DNA Methylation in Cells

DNA methyltransferases (DNMTs) are well-established key players in epigenetic regulation, specifically as cytosine methylases. These enzymes are pivotal for cell proliferation and differentiation, as they facilitate the transfer of a methyl group from the ubiquitous methyl donor S-adenosyl-L-methionine (SAM) to the 5-position of cytosine residues [33]. Our study focused on assessing the levels of DNMT, 5-methylcytosine (5mC), SAM, and S-adenosyl-L-homocysteine (SAH), the latter being the end-product of methylation reactions in the body, all of which serve as indicators of methylation.

**Figure 2:**
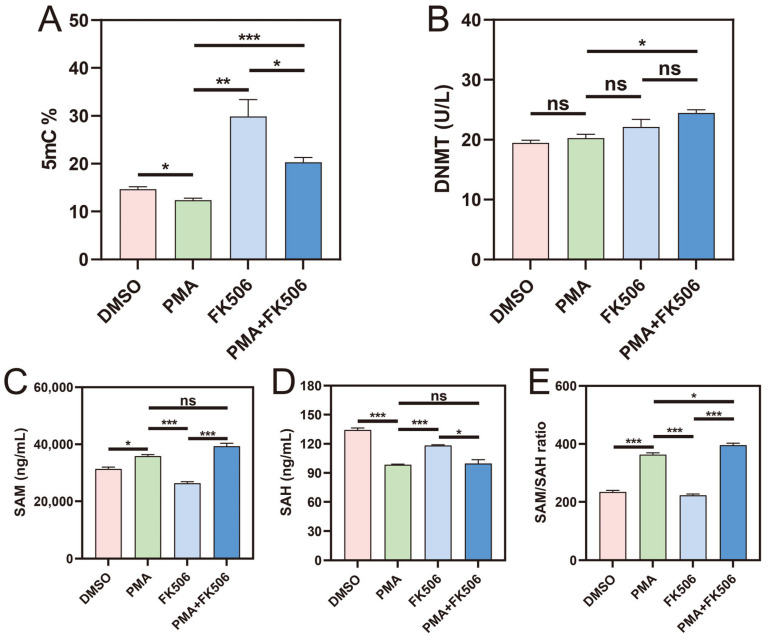
The activity of DNMT, 5mC%, SAH and SAM contents, and SAM/SAH ratio in the four groups of cells. The level of DNA methylation is presented via (**A**) 5mC%, (**B**) DNMT enzyme activities, (**C**) SAM content, (**D**) SAH content, (**E**) SAM/SAH ratio. ns indicates no significant difference, * indicates *p* < 0.05, ** indicates *p* < 0.01, *** indicates *p* < 0.001. n = 3.

Significantly, the percentage of 5mC, a marker of methylation levels, exhibited a notable increase following FK506 treatment and a substantial decrease after PMA + FK506 treatment (Figure 2A). The levels of DNMT remained unaltered upon treatment with PMA alone (Figure 2B), yet the percentage of 5mC displayed a marked decrease (Figure 2A). To delve deeper into the methylation process, we scrutinized the levels of SAM (the methyl donor) and SAH (the methyl receptor). Following PMA treatment, SAM displayed a substantial increase (Figure 2C), while SAH showed a significant decrease (Figure 2D). Consequently, the SAM/SAH ratio exhibited a noteworthy increase (Figure 2E), signifying a reduction in methylation levels induced via PMA. Contrarily, after FK506 treatment, SAM significantly decreased, SAH significantly increased, and the SAM/SAH ratio significantly decreased, indicative of an increase in methylation levels. These findings align with the changes observed in the percentage of 5mC.

### 3.3. FK506 Changed the Expression Level of DNA-Methylation-Related Genes

**Figure 3:**
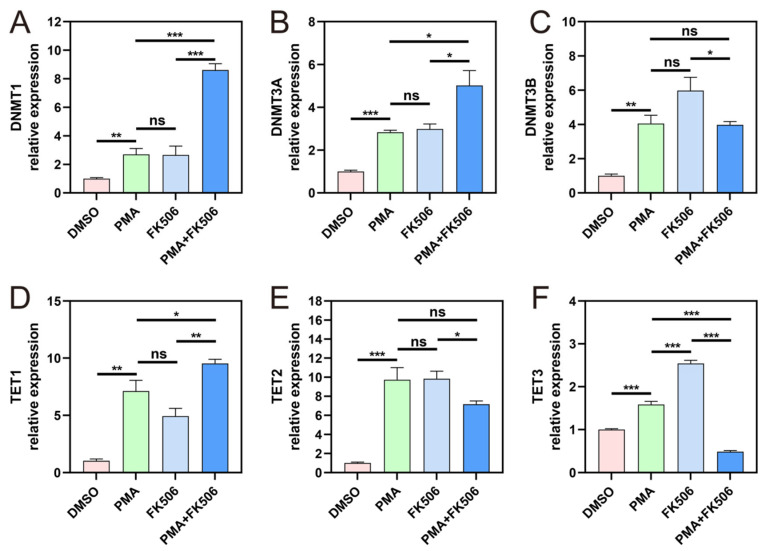
The gene expression of DNMTs and TETs in the four groups of cells. The gene expression levels of (**A**) DNMT1; (**B**) DNMT3A; (**C**) DNMT3B; (**D**) TET1; (**E**) TET2; (**F**) TET3. ns indicates no significant difference, * indicates *p* < 0.05, ** indicates *p* < 0.01, *** indicates *p* < 0.001. n = 3.

DNA-methylation-related genes include DNA methyltransferases (DNMTs), TETs, and DNA demethylation genes. DNMTs and TETs play pivotal roles in governing dynamic changes in DNA methylation [33]. The DNMT family comprises four members, namely, DNMT1, DNMT3A, DNMT3B, and DNMT3L. Upon PMA treatment, the expression levels of DNMT1, DNMT3A, and DNMT3B experienced significant increases, with no significant distinction between them and those after FK506 treatment, suggesting that PMA and FK506 could activate the expression of these three DNMT genes in a similar manner. However, when neutrophil cells were concurrently treated with PMA and FK506, the expression of DNMT1 and DNMT3A genes significantly increased (Figure 3A,B), while DNMT3B gene expression decreased to some extent (Figure 3C). This may indicate that enzymes within the DNMT family possess distinct regulatory functions while maintaining methylation at a relatively stable level.

Ten eleven translocation protein 1 (TET1) holds the capability to modify methylcytosine and potentially erase DNA methylation [34]. TET1 is a member of a trio of proteins, in addition to TET2 and TET3, that catalyze the successive oxidation of 5-methylcytosine (5mC) to 5-hydroxymethylcytosine (5hmC), 5-formylcytosine (5fC), and 5-carboxylcytosine (5caC) [35]. In our analysis, we observed significant increases in TET1, TET2, and TET3 following PMA and FK506 treatment, with TET3 showing a notably more pronounced increase after FK506 treatment (Figure 3D–F). However, when subjected to PMA + FK506 treatment, TET1 exhibited an increase relative to treatment alone (Figure 3D), while TET2 decreased (Figure 3E), particularly TET3 (Figure 3F). This suggests that FK506 may aid in mitigating a PMA-induced decrease in methylation levels. Considering the changes in DNMT and TET genes, it is evident that both PMA and FK506 significantly activate the expression of methylated genes. The combination of PMA and FK506 treatment resulted in altered gene expressions, with DNMT1 and DNMT3A increasing, DNMT3B decreasing, TET1 increasing, and TET2 and TET3 decreasing significantly. These findings underscore the capacity of FK506 to modulate the expression of DNA-methylation-related genes, including DNMT and TET genes.

In the combined PMA and FK506 treatment, DNMT and 5mC levels increased, SAM levels surged, and SAH levels remained unaltered. Remarkably, the SAM/SAH ratio exhibited a significant increase compared to the PMA group. These outcomes suggest that PMA diminishes methylation levels through inhibition, while FK506 elevates methylation levels by augmenting the availability of SAM, the methyl donor, in HL-60 cells.

### 3.4. DNMT and TET Protein Levels Were Significantly Up-Regulated via FK506 in HL-60 Cells

**Figure 4:**
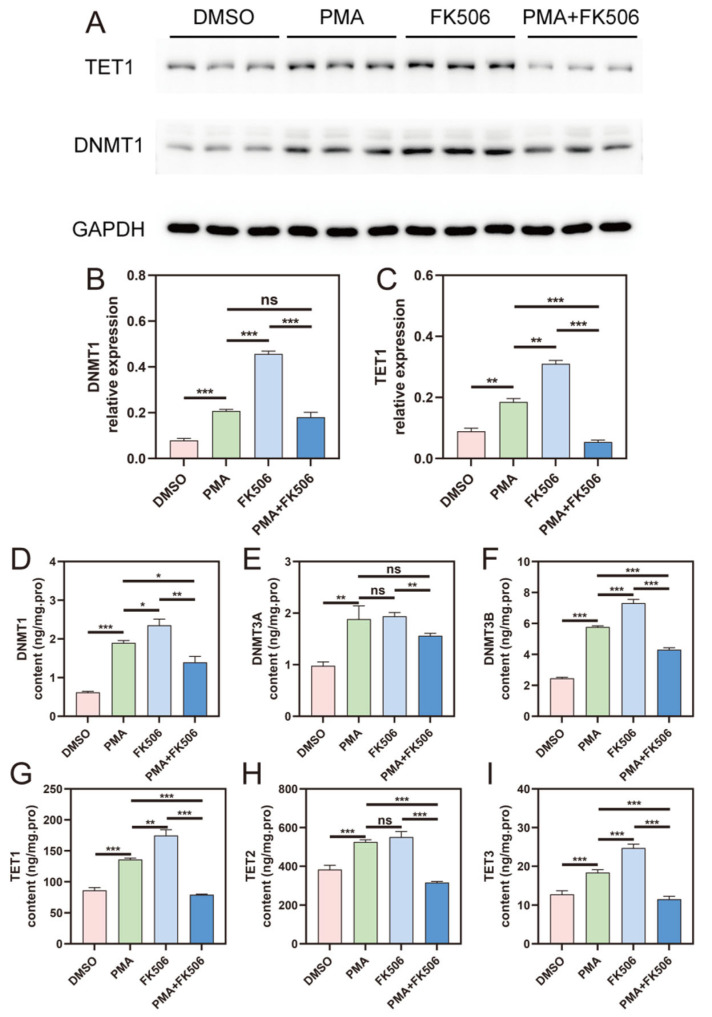
The gene expression of DNMTs and TETs in the four groups of cells. (**A**) Western blot picture of TET1 and DNMT1 in the four groups of cells. Western blot data of (**B**) DNMT1 and (**C**) TET1 expression levels. (**D**–**I**) The protein content of DNMT1, DNMT3A, DNMT3B, TET1, TET2, TET3, respectively. ns indicates no significant difference, * indicates *p* < 0.05, ** indicates *p* < 0.01, *** indicates *p* < 0.001. n = 3.

To further investigate the expression of DNMT and TET genes, we detected the levels of these DNA-methylation-related proteins in cells. The expression levels of DNMT1 and TET1 were assessed using a Western blot analysis (Figure 4A), and the data are plotted in Figure 4B,C, respectively. ELISA kits were employed to measure the levels of DNMT1, DNMT3A, DNMT3B, TET1, TET2, and TET3 proteins (Figure 4D–I), respectively. The results revealed that FK506 treatment could activate the expression of DNMT1 and TET1, as evidenced by the increased protein levels of DNMT1 and TET1 (Figure 4B,C). However, FK506 did not affect the expression of DNMT3A (Figure 4D). Significantly, the protein levels of DNMT3B and TET3 were found to be increased (Figure 4F,I). These findings substantiate the notion that FK506 enhances the DNA methylation process within cells by up-regulating the expression of key DNA-methylation-related proteins, including DNMT1, DNMT3B, TET1, and TET3. This enhancement may contribute to the suppression of NET formation in HL-60 cells.

### 3.5. Correlation Analysis among Genes, Proteins, and DNA Methylation

**Figure 5:**
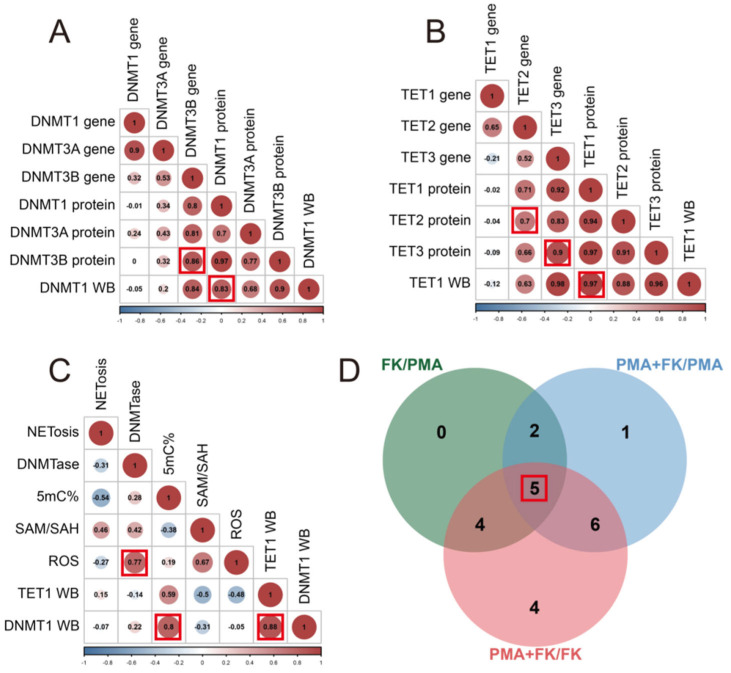
Correlation and Venn analyses. (**A**) Correlation analysis among DNMT genes and proteins. The numbers in the circles are correlation coefficients. The numbers represent the number of different indicators. The larger the number, the stronger the correlation. The color and size of the circles indicate the magnitude of the correlation. If the color is blue, this indicates a stronger negative correlation. If the color is red, this indicates a stronger positive correlation. (**B**) Correlation analysis among TET genes and proteins. (**C**) Correlation analysis among 5mC% and other data. The numbers in the circles are correlation coefficients. The red box indicates that the correlation coefficient is greater than 0.7 and the number of indicators with the same difference on A, B and C figures. (**D**) Venn analysis of three comparisons (FK/PMA, FK + PMA/PMA, FK + PMA/FK). The red box indicates the number of indicators with the same difference.

We conducted a correlation analysis on the differential data, uncovering significant positive and negative correlations between genes and proteins. A correlation coefficient was considered significant when surpassing 0.6 (indicating a positive correlation) or falling below −0.6 (indicating a negative correlation). Notably, a strong positive correlation was evident, with a correlation coefficient of 0.86, between the DNMT3B gene and protein expression. Similarly, DNMT1 protein expression and content exhibited a positive correlation with a correlation coefficient of 0.83 (Figure 5A, DNMT1 WB). The TET2 gene and protein expression also displayed a positive correlation, marked by a correlation coefficient of 0.7, while the TET3 gene and protein expression demonstrated a substantial positive correlation, with a higher correlation coefficient of 0.9. Additionally, the protein expression and content of TET1 revealed a robust positive correlation, with a correlation coefficient of 0.97 (Figure 5B, TET1 WB). Furthermore, a positive correlation emerged between ROS and DNMTase, exhibiting a correlation coefficient of 0.77. Further analysis unveiled a positive correlation between DNMT1 expression (DNMT1 WB) and 5mC% levels, marked by a correlation coefficient of 0.8, while TET1 expression (TET1 WB) exhibited a positive correlation, with a correlation coefficient of 0.88 (Figure 5C).

Upon scrutinizing the differential data from the three PMA, FK (FK506), and FK + PMA treatment groups, we observed significant changes in five data points among the three groups. These data points encompassed 5mC% levels, TET3 gene expression, TET3 protein content, DNMT3B protein content, and TET1 protein expression (TET1 WB) (Figure 5D). This indicates that methylation alteration is a critical response of neutrophils to FK506, as manifested by the changes in 5mC% levels, the key gene for methylation (TET3), and the increase in DNMT3B protein content, signifying enhanced methylation.

### 3.6. Diagram of FK506 Reducing NET Formation by Enhancing DNA Methylation

**Figure 6:**
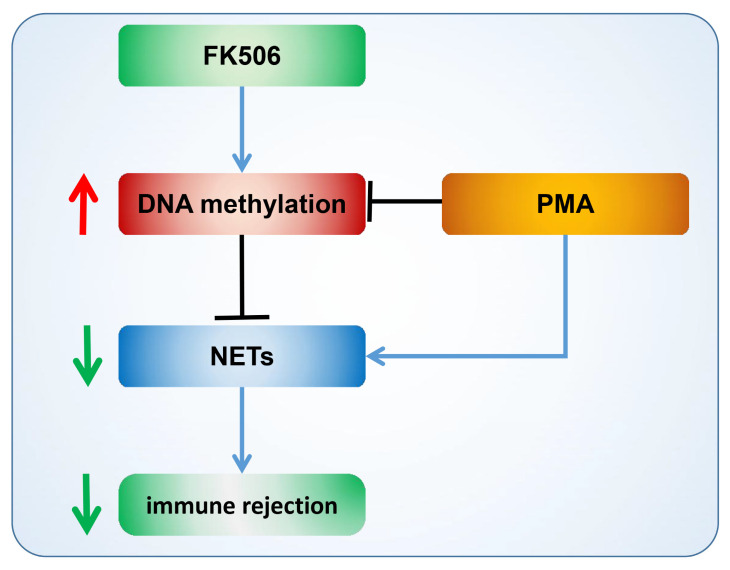
Diagram of FK506 regulation of NETs and immune rejection via DNA methylation. The arrows indicate promotion, the horizontal line indicates inhibition, and the downward green arrows indicates a decline, the upward red arrows indicates an increase.

Through a meticulous analysis of the aforementioned experimental results, we have constructed a comprehensive mechanism diagram (Figure 6) to shed light on the underlying processes. Our findings uncover that PMA, a potent stimulant, possesses the remarkable capacity to induce substantial NET formation, hinting at a potential connection between PMA and the inhibition of DNA methylation, a critical epigenetic modification. Notably, our study also unveils the divergent effects of FK506, a widely utilized immunosuppressive drug, on NET formation. We have observed that FK506 exhibits a pronounced capability to curtail NET production, suggesting its potential to ameliorate immune rejection. The mechanism underlying this effect seems to revolve around the FK506-mediated enhancement of DNA methylation. Specifically, this immunosuppressive agent triggers the up-regulation of key DNA-methylation-related genes and proteins, including DNMT3B, TET1, and TET3. These elements collaborate to bolster DNA methylation, consequently leading to a reduction in NET formation and immune rejection. This intricate interplay between FK506 and the DNA methylation process carries significant implications for immune modulation, rendering it a promising therapeutic target for alleviating immune-rejection-related complications across diverse cellular contexts.

## 4. Discussion

In the past, the mechanism by which immune rejection occurs in the context of NETs was unclear, but this process is highly important. By focusing on the analysis of FK506 primer DNA methylation changes, we clarified that the occurrence of NETs is closely related to methylation, which is consistent with the results published in a 2020 study [36]. Here, we treated neutrophils with the immunosuppressive drug FK506 and found that it can significantly change DNA methylation levels, which, in turn, affect the levels of NETs. This may be a reason for FK506’s action as an immunosuppressive agent.

Since NETs were discovered in 2004 [37], numerous studies have demonstrated the mechanism of NET formation and their function in innate immunity and inflammation. NETs play an antimicrobial role, but excessive NETs are harmful and can cause inflammation and tissue damage [38]. Therefore, changes in NETs will involve the occurrence of many diseases and physiological processes. FK506 significantly increases DNA levels and suppresses the generation of NETs (NETosis was significantly decreased, as shown in Figure 1H). The reason for this has not been discovered. It is possible that NETs could be formed during the process of organ transplantation [39]. NETs are structures that are formed via neutrophils in response to infection or inflammation. However, the release of NETs can also cause tissue damage, and excessive or inappropriate NET formation has been implicated in the development of autoimmune and inflammatory diseases. It is possible that NETs could be formed in response to the stress of surgery or because of inflammation or infection during the transplantation process [40,41,42,43,44,45,46]. More research is needed to understand the role of NETs in organ transplantation and the potential consequences of NET formation.

There is some evidence to suggest that DNA methylation, a process that involves the addition of a methyl group to cytosine residues in the DNA molecule, can regulate NET formation. Researchers have found that DNA methylation was increased in neutrophils from individuals with autoimmune diseases and that this increase in methylation was correlated with an increased NET formation. It remains to be determined whether procainamide and hydralazine also inhibit DNA methylation in neutrophils, similar to as described in T cells, and whether this might induce neutrophil DNA demethylation. Demethylated DNA released within NETs might result in enhanced TLR-9 stimulation, as TLR-9 is sensitive to demethylated DNA [47]. In 2020, Yasuda, H. et al. found that DNA demethylation increases NETosis, which was increased by the addition of the DNMT inhibitor 5-azacytidine (Aza), and genomic DNA in Aza-stimulated neutrophil-like cells was demethylated [36]. However, the relationship between DNA methylation and NET formation is not fully understood, and more research is needed to determine the exact mechanisms by which DNA methylation may regulate NET formation and the potential therapeutic use of drugs that modulate DNA methylation as a way to regulate NET formation in different disease states. We studied the methylation levels of cells treated with FK506 and found that FK506 can significantly increase the DNA methylation levels (5mC%) of cells (Figure 2A). Simultaneously, the methylation donors (SAM) and methylation receptors (SAH) related to DNA methylation, as well as the SAM/SAH ratio (Figure 2C–E), all underwent significant changes, indicating that FK506 significantly enhanced DNA methylation levels. This finding may answer the question of why FK506 increases NET suppression: FK506 increases DNA methylation levels, thereby stabilizing DNA’s structure, making DNA less prone to degradation, and rendering it harder to form NETs, thus reducing NET levels.

To further confirm the occurrence of methylation regulation, we examined the expression of DNA-methylation-related genes and proteins within cells. We observed significant alterations in the gene expression of DNMT (Figure 3A–C) and TET (Figure 3D–F) families, indicating that FK506 can impact the expression of methylation-related genes, thus regulating the methylation process. At the protein level, DNMTs and TETs (Figure 4) also exhibited substantial changes following FK506 treatment. By means of correlation and Venn analyses, DNMT3B and TET3 emerged as potential pivotal factors in DNA methylation. Epigenetic mechanisms, particularly DNA methylation, play a significant role in neutrophil differentiation and NET formation. The inhibition of DNMT1, which is responsible for DNA demethylation, triggers ROS-independent NETosis [48]. Our investigation unveiled a robust correlation between methylation and the substantial changes induced via FK506. These findings align with previous research results.

Therefore, we believe that FK506 maintains the neutrophil balance by elevating DNA methylation levels, thereby stabilizing DNA’s structure and reducing NET generation, thus reducing immune rejection reactions. Our study has offered compelling evidence that suggests FK506 can influence NETs through methylation regulation. Nonetheless, a direct link between FK506 and the methylation process remains to be definitively established. The precise mechanism by which FK506 influences the methylation process remains unknown. In forthcoming research, we intend to employ transcriptome sequencing techniques to identify genes whose methylation is inhibited by FK506. Through the analysis of these genes, we aim to unravel the causal connection between FK506 and the methylation process. Furthermore, we will investigate the involvement of the one-carbon pathway, with a particular focus on the methylation donor S-adenosylmethionine (SAM), to gain deeper insights into the mechanisms through which FK506 mediates immunosuppression. By elucidating the mechanism underpinning the immunosuppressive effects of FK506 and providing robust evidence, we aspire to offer valuable insights for clinical applications. This forthcoming research will address current knowledge gaps and advance our comprehension of how FK506 modulates methylation and its implications in immunosuppression.

## 5. Conclusions

In conclusion, our study has provided novel insights into the immunomodulatory effects of FK506 by elucidating its role in inhibiting the production of NETs. Importantly, we have identified DNA methylation enhancement as a key mechanism underlying this effect, thereby uncovering a previously unrecognized aspect of FK506’s immunosuppressive action. The significance of our findings lies in the potential clinical implications they hold. By elucidating this novel mechanism, our study has the potential to guide future research in the fields of immunosuppression and organ transplantation. The ability of FK506 to modulate NET formation through DNA methylation highlights its promising role in reducing immune rejection and improving transplantation outcomes. Taken together, our study not only unravels a previously unexplored mechanism of action for FK506, but also offers a valuable direction for future clinical investigations and strategies in the realm of organ transplantation. The discovery of this mechanism, along with its potential for minimizing side effects while augmenting transplant outcomes, underscores its considerable clinical worth, necessitating further exploration and validation.

## Figures and Tables

**Table 1:** Primer sequences of the qPCR.

Gene Name	Forward Primer (5′–3′)	Reverse Primer (5′–3′)
*DNMT1*	ACCGCTTCTACTTCCTCGAGGCCTA	GTTGCAGTCCTCTGTGAACACTGTGG
*DNMT3A*	CACACAGAAGCATATCCAGGAGTG	AGTGGACTGGGAAACCAAATACCC
*DNMT3B*	AATGTGAATCCAGCCAGGAAAGGC	ACTGGATTACACTCCAGGAACCGT
*TET1*	TCTGTTGTTGTGCCTCTGGA	CCCATGACCACATCTACTGT
*TET2*	AGCAATAGGACATCCCTGAG	CATCTAGGAGCAGGTCCTAA
*TET3*	CGGATCGAGAAGGTCATCTA	ATGACGATCACAGCGTTCTG
*GAPDH*	CAAGGTCATCCATGACAACTTTG	GTCCACCACCCTGTTGCTGTAG

**Table 2:** Mass spectrometry parameters of MRM in 5mC and C.

Compound	Parent (*m*/*z*)	Daughters (*m*/*z*)	Cone Voltage (V)	Collision Energy (V)	Comments
Cytidine (C)	244.23	112.06	28	10	Quantitative ion pair
	244.23	94.96	28	46	Qualitative ion pair
5mC	258.23	126.11	22	10	Quantitative ion pair
	258.23	108.89	22	42	Qualitative ion pair
Thymidine (IS)	243.23	127.11	16	10	Quantitative ion pair
	243.23	117.06	16	6	Qualitative ion pair

**Table 3:** Mass spectrometry parameters of MRM in SAM and SAH.

Compound	Parent (*m*/*z*)	Daughters (*m*/*z*)	Cone Voltage (V)	Collision Energy (V)	Comments
SAH	385.2	134.1	30	22	Quantitative ion pair
	385.2	136.1	30	20	Qualitative ion pair
SAM	399.2	250.1	30	16	Quantitative ion pair
	399.2	298.2	30	12	Qualitative ion pair

## Data Availability

The datasets used and/or analyzed during the current study are available from the corresponding author upon reasonable request.

## Supplementary Materials

The following supporting information can be downloaded at: https://www.mdpi.com/article/10.3390/life13122253/s1. Figure S1. Changes of cell viability in four groups of cells.

## Abbreviations

DMEM	Dulbecco’s Modified Eagle Medium
DMSO	Dimethyl sulfoxide
DNMT	DNA methyltransferase
ELISA	Enzyme-linked immunosorbent assay
LC MS/MS	Liquid chromatography–tandem mass spectrometry
MTT	3-(4,5-Dimethylthiazol-2-yl)-2,5-diphenyl tetrazolium bromide
NETs	Neutrophil extracellular traps
NETosis	Neutrophil cell-death-associated NETs
PMA	Phorbol myristate acetate
5mC	5-methylcytosine
MRM	Multiple-reaction monitoring
ROS	Reactive oxygen species
SAH	S-adenosylhomocysteine
SAM	S-adenosylmethionine
SE	Standard error
TET	Ten eleven translocation
UPLC	Ultra-performance liquid chromatography
WB	Western blot
