# Supplementary material for: Tacrolimus Maintains the Balance of Neutrophil Extracellular Traps by Inducing DNA Methylation of Neutrophils to Reduce Immune Rejection
Source: Life (Basel). 2023 Nov 25;13(12):2253. doi: 10.3390/life13122253 (PMC10744459; doi:10.3390/life13122253)
Supplement: Supplementary file 1 [file life-13-02253-s001.zip › life-2676451-supplementary.pdf]

Supplementary Materials

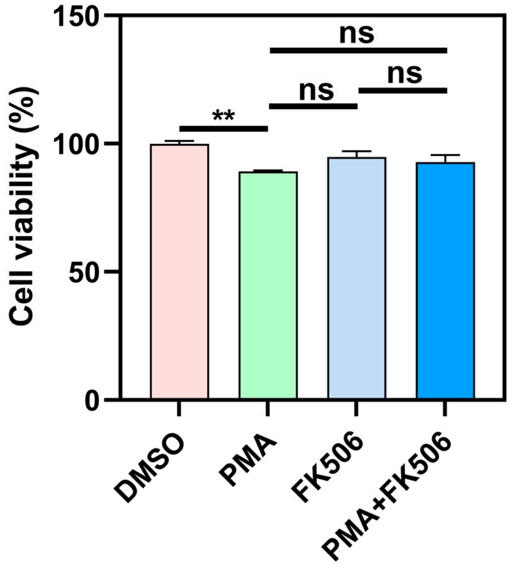

Figure S1. Changes of cell viability in four groups of cells. The figure provides a cell viability analysis of the effects of DMSO, PMA, fk506, and PMA+FK506 treatment. n=3, and statistical significance is denoted as follows: ** for $p < 0.01$, and "ns" to indicate the no significant difference. Data are presented as mean ± SE.
